# Demographic effects of extreme weather events: snow storms, breeding success, and population growth rate in a long-lived Antarctic seabird

**DOI:** 10.1002/ece3.1357

**Published:** 2014-12-23

**Authors:** Sébastien Descamps, Arnaud Tarroux, Øystein Varpe, Nigel G Yoccoz, Torkild Tveraa, Svein-Håkon Lorentsen

**Affiliations:** 1 Norwegian Polar Institute, Fram Centre9296, Tromsø, Norway; 2 Akvaplan-Niva, Fram Centre9296, Tromsø, Norway; 3 University Centre in Svalbardpb 156, 9171, Longyearbyen, Norway; 4 Department of Arctic and Marine Biology, UiT The Arctic University of Norway9037, Tromsø, Norway; 5 Norwegian Institute for Nature Research, Fram Centre9296, Tromsø, Norway; 6 Norwegian Institute for Nature Research7485, Trondheim, Norway

**Keywords:** Antarctic petrel, body condition, extreme event, individual state, offspring survival, *Thalassoica antarctica*

## Abstract

Weather extremes are one important element of ongoing climate change, but their impacts are poorly understood because they are, by definition, rare events. If the frequency and severity of extreme weather events increase, there is an urgent need to understand and predict the ecological consequences of such events. In this study, we aimed to quantify the effects of snow storms on nest survival in Antarctic petrels and assess whether snow storms are an important driver of annual breeding success and population growth rate. We used detailed data on daily individual nest survival in a year with frequent and heavy snow storms, and long term data on petrel productivity (i.e., number of chicks produced) at the colony level. Our results indicated that snow storms are an important determinant of nest survival and overall productivity. Snow storm events explained 30% of the daily nest survival within the 2011/2012 season and nearly 30% of the interannual variation in colony productivity in period 1985–2014. Snow storms are a key driver of Antarctic petrel breeding success, and potentially population dynamics. We also found state-dependent effects of snow storms and chicks in poor condition were more likely to die during a snow storm than chicks in good condition. This stresses the importance of considering interactions between individual heterogeneity and extreme weather events to understand both individual and population responses to climate change.

## Introduction

Ongoing climate change is considered to be a major driver of populations and ecosystems (Intergovernmental Panel on Climate Change 2014). Until relatively recently, most studies about the ecological consequences of climate changes have focused on changes in average temperature. However, there is increasing focus on the importance of climate variability and weather extremes (Jentsch et al. 2007), which may represent one of the most important facets of ongoing climate change.

Most scenarios for future climate change predict an increase in environmental variability, and in the frequency and strength of extreme events (Easterling et al. 2000; Solomon et al. 2007; Smith 2011b), at least at a regional scale (Huntingford et al. 2013). Extreme weather events are generally defined both in statistical terms (i.e., low frequency of occurrence) and in ecological impact (Smith 2011a,b). Using such definitions, weather events must occur rarely (meaning usually an occurrence <5%) to be considered as extreme. This has, however, important limits. Indeed, using this statistical criterion, a given weather extreme may not be extreme anymore if its frequency of occurrence increases in the future, even if its biological impact stays the same or increases. Moreover, using the frequency of occurrence to characterize weather extremes may lead to counter-intuitive classification. For example, events such as “killer tornadoes” would not be considered extremes in some American states because their probability of occurrence is high (e.g., 298 tornados have killed people in period 1880-2005 in Arkansas, meaning an average frequency of occurrence of 2.4/year, Ashley 2007). In this context, an extreme event might be defined as one in which the ability of an organism or population to acclimate is substantially exceeded, with often persistent effects after the event, resulting in longer-term impacts on fitness (Gutschick and Bassirirad 2003). This is the definition we use here.

Extreme events such as heat waves, droughts, or typhoons may have dramatic effects on individuals, populations, and even ecosystems (Allen and Breshears 1998; Jiguet et al. 2006; Frederiksen et al. 2008; Van De Pol et al. 2010; Moreno and Møller 2011; Niu et al. 2014). On a shorter timescale, the impacts of extreme events on ecological systems may be larger than the ones due to changes in averages of weather variables alone (Thompson et al. 2013), but extreme events have received far less attention in ecology to date.

Relatively few studies have documented impacts of extreme weather events on wildlife (see Moreno and Møller 2011 for a review on the effects on life history, and Zimmermann et al. 2009 for an example on the effects on species distribution), mainly because extreme events are by definition rare and their study mainly opportunistic. However, if the frequency and severity of extreme weather events are to increase, there is an urgent need to understand and predict the ecological consequences of such events, including impacts on demographic parameters. Wildlife may be highly vulnerable to extreme weather events (Newton 1998; Parmesan et al. 2000; Frederiksen et al. 2008; Van De Pol et al. 2010) and, due to the low frequency and unpredictability of such events, may have evolved limited adaptations to deal with them. Even if increased climate variability and resulting extreme weather conditions at a global scale have been recently debated (Rhines and Huybers 2013), such increases are supported at a regional scale, and occurrences have been documented in most of North America, Europe, and Antarctica (Huntingford et al. 2013). More specifically, increase in snow storm severity and frequency seems to be already occurring along the Antarctic Peninsula, at least in spring, with important ecological effects, notably on Adélie penguins (McClintock et al. 2008).

Our study focused on the impact of summer snow storms on a long-lived seabird, the Antarctic petrel *Thalassoica antarctica*. Although difficult to quantify, due to the rareness of these events, the effects of snow storms on survival during the most vulnerable stages, such as eggs and chicks, can be drastic and immediate thereby strongly decreasing reproductive success (e.g., Büβer et al. 2004 on Wilson's storm petrel, see also Saether et al. 1997 for anecdotic observations on Antarctic petrels). We aimed at quantifying the effect of snow storms on nest survival (i.e., survival of its egg/chick) in the Antarctic petrel and assessing whether snow storms are an important driver of annual breeding success. We used both detailed data on daily individual nest survival during the 2011/2012 breeding season during which frequent and heavy snow storms occurred, as well as long term data (1985–2014) on Antarctic petrel productivity at the colony level (i.e., total number of active nests estimated after the hatching period).

Then, we investigated individual heterogeneity in the response to extreme weather events (Coulson et al. 2001; Fouillet et al. 2006; Pardo et al. 2013). Understanding variation among individuals in their sensitivity to environmental changes might be of paramount importance to understand the overall population response (Benton et al. 2006) and thus its viability in face of climate changes. In case of snow storms, body condition, and thus energetic reserves, in particular may be an important determinant of an individual's response, with the prediction that individuals with lower body condition would have a lower survival and/or breeding success during the storm. Data on adult body condition were not available to assess such heterogeneity, but we were able to test whether chick body condition was associated with survival during a snow storm.

Finally, we integrated the effects of snow storms on petrel breeding success into population demographic models to determine the potential long term effects of snow storms on population growth rate.

## Materials and Methods

### Study colony and species

The study was carried out at the Svarthamaren Antarctic petrel colony (71°53′S, 5°10′E) in Dronning Maud Land, Antarctica. About 200,000 pairs of Antarctic petrels breed at this colony which is located 200 km from the coast. The Antarctic petrel is a medium-sized petrel that weighs *ca*. 600 g. It breeds on the ground in scree slopes mainly in east Antarctica, and Svarthamaren is the largest known colony (Mehlum et al. 1988; Van Franeker et al. 1999). Nests are densely located (0.8 breeding pairs per m^2^, Mehlum et al. 1988), and often placed close to rocks, which offer varying amounts of shelter (Varpe and Tveraa 2005). Antarctic petrels lay a single egg at the end of November/early December and both parents incubate and feed the chick. For the first 7–15 days following hatching, one parent guards the chick at the nest, while the mate is at sea (Lorentsen and Røv 1995). Hatching occurs around mid-January and fledging in late February/early March. The only predator at the Svarthamaren colony is the south polar skua (*Catharacta maccormicki*), which mainly preys upon eggs and chicks (Brooke et al. 1999, pers. obs.).

### Nest monitoring and nest survival analyses

In the 2011/2012 breeding season, we monitored 358 nests located in four different study plots within the colony. Nests were individually marked with a numbered tag. Nest monitoring started on 5 December and ended up on 18 February, and nests were, on average, visited every 5 days. During this 2011/2012 breeding season, four snow storms occurred (8 December, 16–18 December, 22–24 January, 9–11 February).

Antarctic petrel breeding success was estimated with nest survival analyses (Rotella et al. 2004). We used program MARK (White and Burnham 1999) to estimate daily nest survival and to test hypotheses concerning the effect of snow storms. We used an information theoretic approach to evaluate the performance of a priori models (Burnham and Anderson 2002). We based this model selection on the Akaike information criterion or AIC (Akaike 1973). Models are detailed in Table1. We considered either that all storms had the same effect on daily nest survival, that storms occurring during the same breeding stage (incubation or chick rearing) had the same effect, or that all storms had a different effect. We also considered models where the storm effect on nest survival either started 1 day after the storm began, continued some days after the storm ended, or both. Indeed, it is unlikely that eggs or chicks die at the very beginning of a storm, because adults will unlikely desert their nest at or chicks may have enough energetic reserves to survive through the very beginning of a storm. Therefore, storm effects on nest survival may be negligible in the first day of the storm. Similarly, such effects may carry on after the last day of the storm. Storms may exhaust the birds, and if adults are not quickly replaced by their partner or chicks fed by their parents, nest desertion or chick death may then occur within some lag period after the end of a storm. Here, we only show the results from models which considered that storms impacted nest survival from 1 day after the storms started up to 1 day after the storms ended. Other lag values did not improve the fit of the models and are not shown.

**Table 1:** Model selection for the daily nest survival in the Svarthamaren Antarctic petrel colony (71°53′S, 5°10′E) in season 2011/2012. Nest monitoring (*n* = 358) occurred on 75 days between December 5 to February 18. Four snow storms occurred during that period, two during the incubation period (8 December and 16–18 December) and two during the chick brooding or rearing period (22–24 January and 9–11 February). Details about each model are given in the Materials and Methods section. np represents the number of estimated parameters, Dev the deviance of the model, and *AIC* its Akaike information criterion (calculated as Dev *+ 2 × *np). *ΔAIC* represents the difference in AIC units compared to the model with lowest AIC. *R*^2^ represents the square of the Pearson correlation between estimates from a given model and estimates from the time-dependent model (i.e., model *S(t)*); %Dev represents the proportion of deviance explained by this given model (see Materials and Methods)

#	Model description	Notation	np	Dev	AIC	ΔAIC	*R*^2^	%Dev
1	Constant survival with a snow storm effect/same effect for the 2 “incubation storms” and the 2 “chick rearing” storms/lagged effect^1^	S(.+Storm_1–2, 3–4/lagged_)	3	1407.596	1413.596	0.000	0.30	0.29
2	Constant survival with a snow storm effect/different effect for all 4 storms/lagged effect^1^	S(.+Storm_1, 2, 3, 4/lagged_)	5	1406.566	1416.476	2.880	0.32	0.30
3	Time-dependent survival	S(t)	62	1295.506	1419.506	5.910	1.00	1.00
4	Constant survival with a snow storm effect/different effect for all 4 storms	S(.+Storm_1, 2, 3, 4_)	5	1427.520	1437.520	23.924	0.12	0.17
5	Constant survival with a snow storm effect/same effect for all 4 storms/lagged effect^1^	S(.+Storm_1 – 4/lagged_)	2	1434.125	1438.125	24.529	0.12	0.13
6	Constant survival with a snow storm effect/same effect for the 2 “incubation storms” and the 2 “chick rearing” storms	S(.+Storm_1-2, 3–4_)	3	1441.722	1447.722	34.126	0.05	0.08
7	Constant survival with a snow storm effect/same effect for all 4 storms	S(.+Storm_1–4_)	2	1448.636	1452.636	39.040	0.03	0.04
8	Constant survival	S(.)	1	1454.379	1456.379	42.783	0.00	0.00

1 Model where there is no storm effect in the 1st day of the storm, but there is a storm effect up to 1 day after the end of storm.

The percentage of variance in nest survival explained by snow storms was estimated using two indices. First, we considered the % of deviance explained (%Dev) calculated as 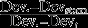 where Dev_storm_ represents the deviance of the model including a storm effect, Dev. represents the deviance of the model where survival was constant, and Dev_*t*_ represents the deviance of the model where survival was time-dependent (Grosbois et al. 2008). We also calculated the square of the Pearson correlation coefficient between survival estimates from the “snow storm models” and survival estimates from the general time-dependent model (Zheng and Agresti 2000).

### Average annual productivity and summer snow storm occurrence

In a second step, we tested for a correlation between the number of storm days per breeding season and the annual number of active nests late after hatching (late January) using 12 years of monitoring data (1985, 1990, 1992–1995, 1997, 1998, 2001, 2012–2014). To estimate Antarctic petrel productivity, we established a grid of 201 square plots of 40 × 40 m (Lorentsen et al. 1993). The center of each plot was marked with an aluminum pole or paint, and in each plot, we counted the number of active nests within a circle of 10 m^2^ (circle of 1.78 m radius centered in the plot). The total number of active nests in the colony was then calculated as: ∑_plot_ (Number of active nests within the 10 m^2^ circle ×160), 160 being the ratio between the surface of the circle and the surface of the plot. Additionally, an estimate of the total number of active nests, posthatch, was obtained in the colony for 1985 (Mehlum et al. 1988) and 1990 (Røv 1991). The methods used for these censuses were the same, but differed from the one used from 1992 onward; they used density estimates from a smaller number of plots (96 plots of 9 m^2^ each on 3 transects) that were extrapolated for the whole colony. These different census methods were comparable, and there is no reason to believe that it could bias the observed trends. Results remain very similar without these two points.

To estimate the number of storm days in years when productivity data were available, we used weather data from the Neumayer research station (70°39′S, 8°15′W), which is located *ca*. 500 km away from the colony. The procedure to validate the use of Neumayer weather data as proxies for the occurrence of storms at Svarthamaren is detailed in Appendix S1. Based on this procedure, we estimated the frequency and duration of all potential storm events at Svarthamaren during the past 32 years.

We used linear regression to assess the relationship between the number of storms per breeding season and the total number of active nests estimated after the hatching period (productivity). Our data indicated a very large variation in annual productivity in years with no snow storms. It is thus clear that Antarctic petrel productivity was strongly affected by other environmental parameters. Oceanographic conditions, and their effects on petrel food availability (i.e. Antarctic krill, Lorentsen et al. 1998), are likely to affect Antarctic petrel breeding success, so that variation in such conditions at sea may confound the effects of snow storms. We thus also considered the effect of snow storms on petrel breeding success while adjusting for the fluctuations in oceanographic conditions by including the average annual value of the Southern Oscillation Index, or SOI (www.cpc.ncep.noaa.gov/) in our models. The SOI is a proxy of oceanographic conditions in the South Atlantic and is linked to Antarctic krill recruitment and dispersal (Murphy et al. 2007). We tested for an SOI effect without or with a time lag of up to 3 years (Murphy et al. 2007).

All computations were completed in software R, using the *lm()* function (R Development Core Team 2010).

### Snow storm and chick body mass

We tested for an effect of chick body mass on the probability to die during a snow storm. We considered chicks weighed in period 2–8 February (*n* = 43), that is, within the week before the storm hit the colony (9–11 February). At this time, chicks were no longer permanently guarded by their parents. We used a logistic regression to assess whether or not chick body mass affected the probability to die during the snow storm. Chick survival was assessed on the day following the storm (12 February); two chicks among the 33 still alive on 12 February died between 14 and 16 of February. This could potentially be due to a delayed effect of the snow storm; however, whichever status was attributed to these two chicks (either “survived” or “died”), results remained the same. Date of measurements, chick age (days), and body size (bill length) may be associated with body mass and confound the association between mass and survival. They were thus included in the model.

All analyses were completed in software R (R Development Core Team 2010) using the *glm()* function with a binomial distribution and a *logit* link function.

### Snow storm and Antarctic petrel long-term stochastic growth rate

The life cycle of Antarctic petrel is poorly known. However, the life cycle of the snow petrel (*Pagodroma nivea*) is well described (Barbraud et al. 2011), and this species is closely related to the Antarctic petrel (Nunn and Stanley 1998). Moreover, average adult survival of Antarctic petrel equals 0.92 (unpublished data), which is close to snow petrel adult survival (0.93; Barbraud et al. 2011). Based on this, we assumed the Antarctic petrel life cycle to be comparable to that of the snow petrel.

**Figure 1:**
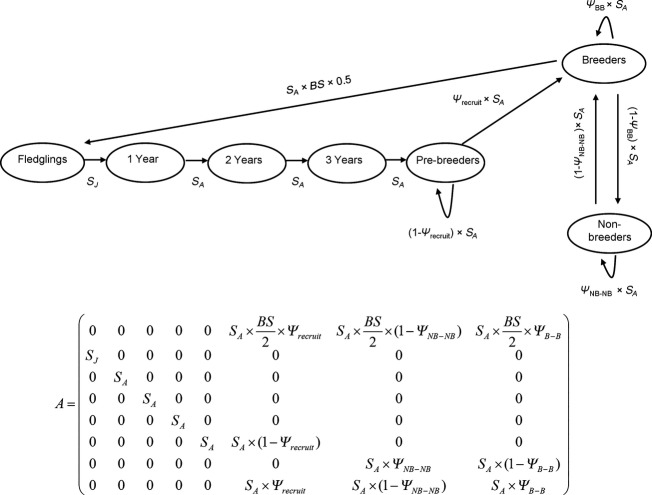
Hypothetical life cycle of the Antarctic petrel. The population matrix *A* contains the vital rates and projects the population from time *t* to *t *+* *1. *BS* represents the average breeding success calculated as the probability that the egg survives from laying to fledging. *S*_*A*_ represents adult survival (survival from 1 year of age onwards) and *S*_*J*_ the average annual survival of juvenile between fledging and 1 year of age. Symbol *Ψ* represents transition probabilities between breeding status “breeder” (B) and “nonbreeder” (NB); *Ψ*_recruit_ represents the probability to breed for the first time.



To gain insight into the potential effects of snow storms on Antarctic petrel population dynamics, we modeled the effects of snow storms on the long-term stochastic growth rate of a population characterized by a snow petrel life cycle (Fig.1). This life cycle is based on delayed first breeding, that occurs at 5 years of age, and on six age classes: fledglings, juveniles (4 classes, from 1 to 4 years of age), and adults (≥5 years old). It also distinguishes between breeders and nonbreeders for adult individuals. The population matrix *A* (Fig.1) projects the population vector *n* that gives the number of individuals in each age class from time *t* to *t + 1*: *n*_*t+1*_ = *A n*_*t*_. To examine the long-term effects of snow storm events on Antarctic petrel population dynamics, we determined the maximum likelihood estimator of the stochastic growth rate with the formula:

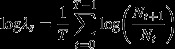
1 (Caswell 2001).

Due to computational issues, we could not set up T at values >10,000 time steps which was not enough to achieve convergence. Therefore, we ran 10 simulations with *T* = 10,000 and average the estimated values of log *λ*_s_.

We considered different situations where the average annual number of storm days per season varied from 0 to 10 days, and with an interannual frequency varying from 0.1 (i.e., storms occur once per decade on average) to 1 (i.e., storms occur every year). At each time step, demographic parameters could vary randomly around their average value to represent the stochastic variations in the environment other than snow storms. Average parameter estimates come from Barbraud et al. (2011) and the standard deviations used in our models from Barbraud, pers. comm.. At each time step, each demographic parameter value was sampled from a beta distribution characterized by the above-defined mean and standard deviation. We modeled two different situations: first, we considered the extreme situation where the storm effect on breeding success was the strongest observed during our study. The strongest storm effect occurred between the 16th and 18th of December 2011, and daily nest survival during this storm was estimated at 0.804. Then, in the second situation, we also considered the average snow storm effects observed in season 2011/2012 where four storms occurred; average daily nest survival during the four snow storms was estimated at 0.893.

All simulations were performed in software R (R Development Core Team 2010).

## Results

### Summer snow storm and breeding success

**Figure 2:**
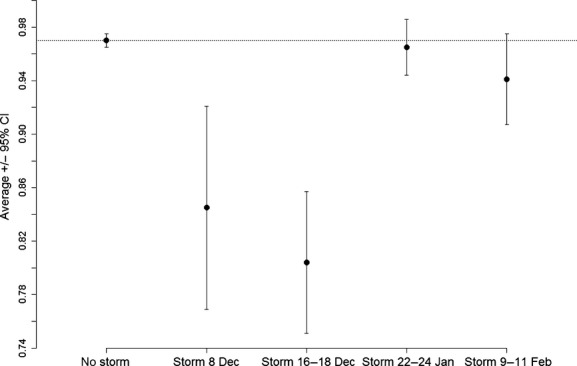
Average nest survival for Antarctic petrels breeding at the Svarthamaren colony, Dronning Maud Land, Antarctica in season 2011/2012 during and excluding the four snow storm events. Estimates are from model S(.+Storm_1, 2, 3, 4/lagged_) (see Materials and Methods and Table1 for details). Dotted line represents the average nest survival outside the stormy periods.

During the 2011/2012 breeding season daily nest survival, estimated using a constant model, was equal to 0.958 (95% confidence interval: [0.954, 0.963]; model *S(.)*, Table1). This corresponded to a breeding success of 4% during the study period (5 December to 18 February), which covers most of the breeding season. The time-dependent model *S(t)* gave a better fit than the constant survival model (Table1), indicating significant temporal variation in nest survival during the season. The model that considered variation in nest survival during the snow storms performed considerably better than the model with constant survival (models 4 vs. 8; Table1). This indicates that daily nest survival was significantly lower during the snow storms (95% confidence: [0.913, 0.949] during the storms versus [0.958, 0.969] outside the storms). Interestingly, models with a storm effect starting only on the second day of the storm but lasting 1 day after the storm end received considerably higher support (models 1 and 2 in Table1). These models showed a lower nest survival during the storms (Fig.2) and explained ∼ 30% of temporal variation in daily nest survival (Table1). All storms did not have the same effect on nest survival (e.g., model 2 vs. 5; Table1; Fig.2), and those differences were mainly due to a different effect in the incubation vs. brooding/rearing periods (model 1 vs. 2; Table1; Fig.2). The snow storms with the most severe impact on nest survival occurred during the incubation period.

Without the snow storms, the average breeding success during the study period would have been between 7 and 15% (estimated from a daily nest survival of 0.965 and 0.975, the lower and upper limit of the 95% confidence interval of nest survival outside the storm events; model 1, Table1). This low value suggests that factors other than snow storms contributed to the very low breeding success of Antarctic petrels in season 2011/2012.

### Annual productivity and number of days with storm events

Annual productivity of the whole Svarthamaren colony was negatively affected by the number of snow storm days per breeding season, but the evidence was not strong (*R*^2^ = 0.30, *t* = −2.06, *P* = 0.067). This regression model predicted an average loss of *ca*. 12,000 nests per day during a snow storm. Assuming a colony size of 180,000 breeding pairs (estimated population size in 2012/2013), this corresponds to an average loss of 7% per storm day.

**Figure 3:**
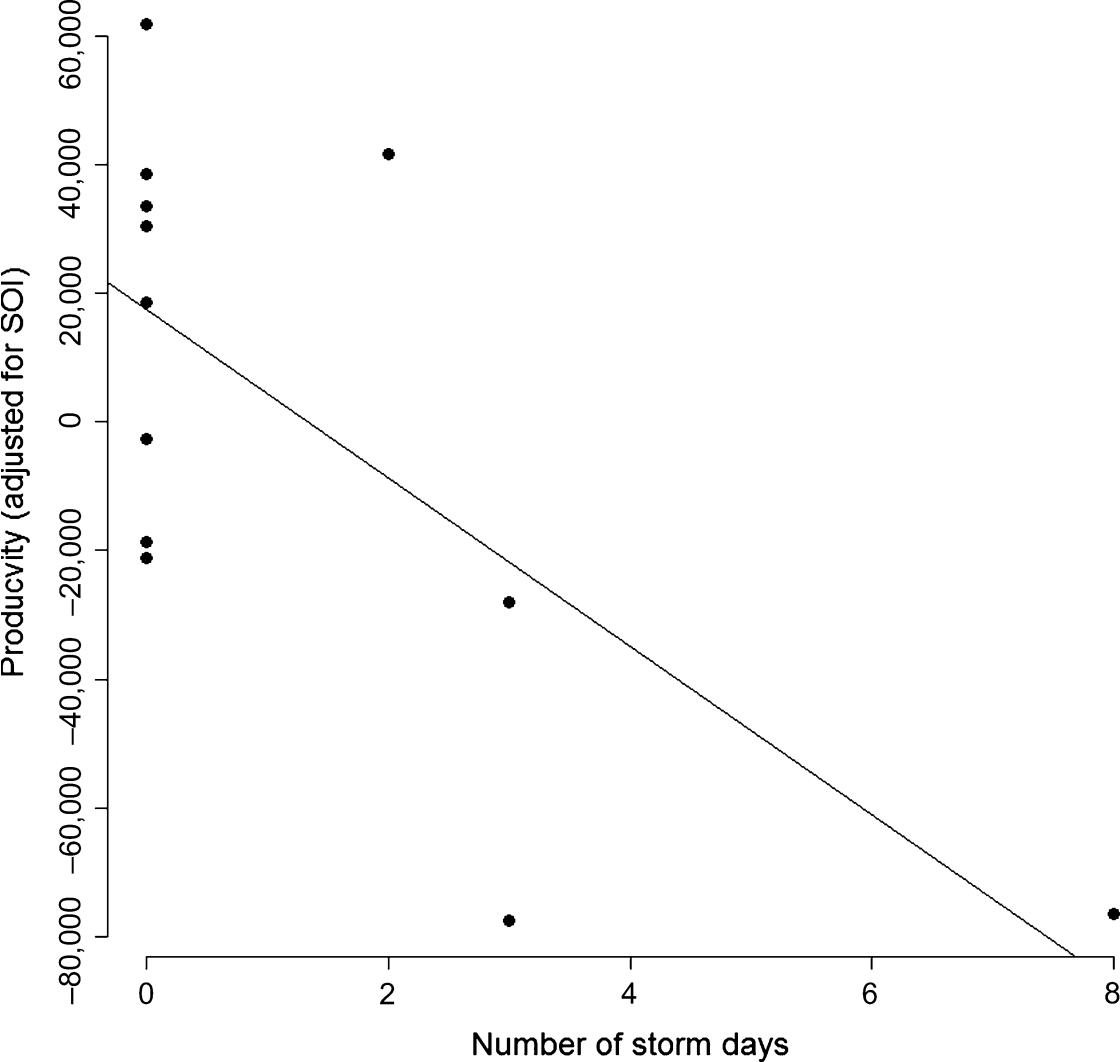
Relationship between annual productivity at the Svarthamaren Antarctic petrel colony, Dronning Maud Land, Antarctica and the number of days with severe storm events per breeding season estimated based on records of weather data (wind speed and atmospheric pressure) from Neumayer Station. The procedure to estimate the frequency of occurrence of severe storm events is detailed in Appendix S1. Annual productivity represents the number of active nests (in thousands) estimated for the whole colony after peak hatching (estimated between mid- and end of January in years 1985, 1990, 1992–1995, 1997, 1998, 2001, 2012–2014; see Materials and Methods and Appendix S1 for details) and adjusted for large-scale oceanographic conditions (see Results). The continuous line represents the predicted number of active nests at the end of January.

When adjusting the productivity for large-scale oceanographic conditions (as measured by the Southern Oscillation Index), the effect of the number of storm days became highly significant (Fig.3). Our final model included both SOI with a lag of 2 and 3 years (Slope _SOI-lag 2_ = −4883 ± 1223 SE, *P* = 0.004 and Slope _SOI-lag 3_ = 3958 ± 1516 SE, *P* = 0.031, respectively); the effect of the number of storm days became highly significant (Slope_snow storms_ = −18372 ± 4530 SE, *P* = 0.003). The overall model explained 78% of the variance in productivity. The effect of the number of storm days remained significant (*P* = 0.034) even when the season 2011/2012, characterized by an extreme number of storm days (*n* = 8), was removed.

### Summer snow storm and chick body mass

**Figure 4:**
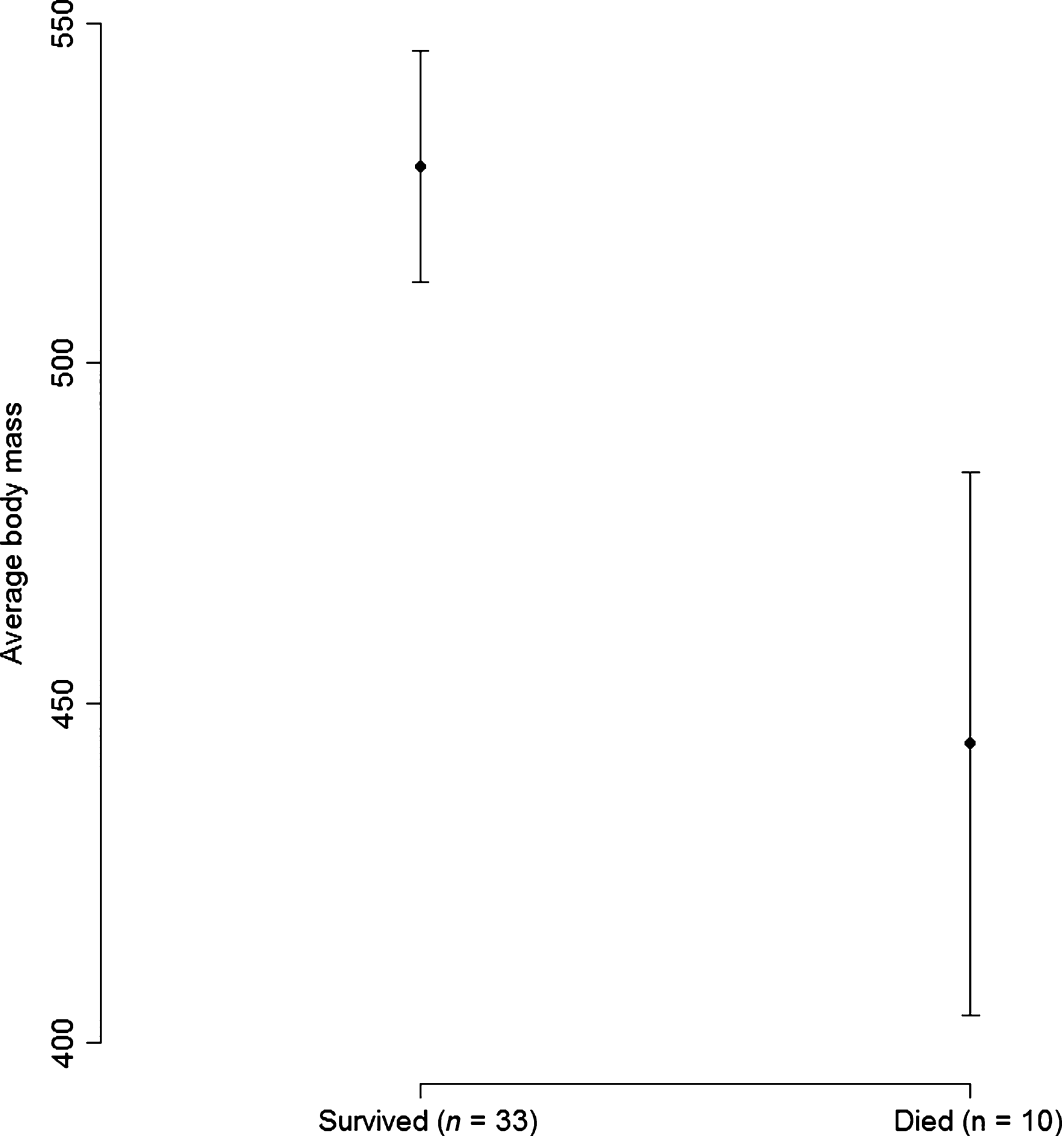
Average (±SE) body mass of chicks that survived and that died during the snow storm that hit the colony after chick independence (storm in period 9–11 February 2012), Svarthamaren Antarctic petrel colony, Dronning Maud Land, Antarctica.

Chick body mass before the storm hit the colony was negatively associated with the probability to die during the storm (*z* = −2.0, *P* = 0.044). Chicks that died during the storm (*n* = 10) weighed on average 85 g less than chicks that survived (*n* = 33; Fig.4), which represents *ca*. 15% of the average chick body mass. Date of measurements, chick age, and body size (bill length) did not significantly affect the probability of dying (*P* > 0.1).

### Summer snow storm and long-term stochastic growth rate

**Figure 5:**
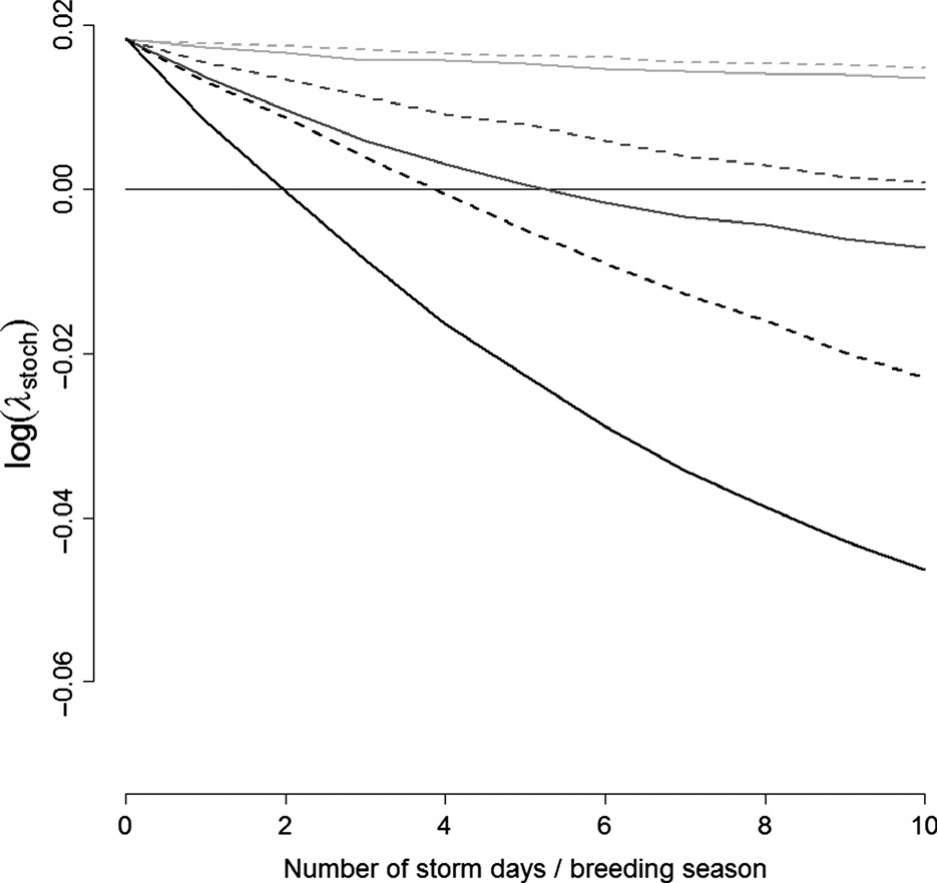
Viability of the Antarctic petrel colony at Svarthamaren, Dronning Maud Land, Antarctica, as a function of the number of storm days per breeding season. Lines represent the long-term stochastic growth rate for different interannual frequencies of storm events (black lines: storms occur every year, i.e., frequency of 1; dark gray lines: storms occur every other year, i.e., frequency of 0.5; light gray lines: storms occur once per decade, i.e., frequency of 0.1). The solid lines represent the effect of severe storms leading to a daily nest survival of 0.804; the dashed lines represent the effect of storms of average severity leading to a daily nest survival of 0.893.

Our simulations indicated that snow storms negatively affect the long-term stochastic growth rate of a population with life cycle described in Fig.1, and those effects depend on the severity, interannual frequency, and duration of the storms (Fig.5). In our example, when storms occur annually (i.e., frequency of 1), one additional storm day per breeding season is, on average, associated with a decline of 0.4 to 0.6% in the long-term stochastic growth rate depending on the severity of the snow storms. In such circumstances, an average of more than 2 severe storm days per breeding season is predicted to be unsustainable over the long term (Fig.5). When considering moderate storms, this threshold increased to an additional 4 storm days on average per breeding season (Fig.5). When storms occur on average every other year (frequency of 0.5), more than five storm days per breeding season in case of severe storms or more than ten days per breeding season in case of moderate storms are unsustainable over the long term (Fig.5).

## Discussion

### Snow storm and Antarctic petrel breeding success

Snow storms appear to be a very important driver of Antarctic petrel breeding success, and potentially of their population dynamics. Our results indicate that snow storms are an important determinant of nest survival and overall productivity at the colony level. Indeed, 30% of the daily nest survival within the 2011/2012 season and nearly 30% of the interannual variation in colony productivity could be explained by snow storm events. Similarly, snow storms were the most important determinant of Wilson's storm-petrels (*Oceanites oceanicus*) breeding success on South Shetland Islands (Büβer et al. 2004), and snowfalls were an significant driver of snow petrel breeding success and proportion of pairs breeding each year (Chastel et al. 1993). Furthermore, Van Franeker et al. (2001) reported that snow drifts had an important detrimental effect on Antarctic petrel breeding success on Ardery Island but indirectly through an effect on predation.

Other examples exist where snow storms negatively impacted breeding success, ranging from passerines (Hendricks and Norment 1992; Decker and Conway 2009) to shorebirds (Sagar et al. 2000) and mammals (Neuhaus et al. 1999). Among seabirds, most of the reported effects of climate fluctuations suggest an indirect effect through the food chain (e.g., Jenouvrier et al. 2003; Sandvik et al. 2005; Hedd et al. 2006; Smith and Gaston 2012). This however may simply represent the dominant tendency of looking at changes in the average temperature in most of the climate-oriented ecological studies and not at specific weather components, such as the extreme events. Increases in temperature (and in particular sea temperature) are indeed likely to affect seabirds via the food chain and not via direct effects on reproduction or survival. However, this does not necessarily apply to extreme weather events. In such cases, like the snow storm effects on Antarctic petrels, direct effects of weather conditions on avian vital rates may be the most important ones and should not be overlooked.

### Snow storms and population viability

Our results emphasize the direct consequences of snow storms on Antarctic petrel breeding success. In long-lived species such as Procellariforms, population growth rate is more sensitive to changes in adult survival than in breeding success (Saether and Bakke 2000). However, large variation in reproductive success can have the greatest influence on population growth rates, even in a long-lived species (Gaillard et al. 2000).

Our simulations indicate that the observed effects of snow storms on Antarctic petrel breeding success have the potential to strongly decrease the long-term stochastic growth rate of the population. These simulations are based on a hypothetical life cycle for the Antarctic petrel and should be taken with caution. However, it is very plausible that the Antarctic petrel life cycle is similar to this hypothetical life cycle as closely related species (i.e., the southern fulmar and snow petrel, Nunn & Stanley) have similar life cycles (Jenouvrier et al. 2005; Barbraud et al. 2011). Therefore, extreme weather events, such as snow storms, affecting seabird breeding success represent an important process to take into account to help understand and predict seabird populations’ responses to ongoing climate changes.

### Intraseasonal timing of the snow storms

Our results suggest that the timing of snow storms may be important in determining nest survival and breeding success; in the 2011/2012 breeding season, storms occurring during incubation had a larger impact on nest survival than storms occurring during chick rearing. One explanation could be related to the structure of the incubation shifts. In December, when two storms hit the colony, many parents on the nest had been incubating, and thus fasting, for many days or weeks (Lorentsen and Røv 1995). Within an evolutionary perspective, long-lived individuals should prioritize their own survival over their current reproductive success (Saether and Bakke 2000), so that adults will abandon their nest if their own survival is compromised. Fasting individuals might not be able to cope with the extra energetic demand caused by a snow storm, which would force them to desert their nest and engage in foraging activities. However, several potentially confounding factors should be considered.

First, the average severity of the storms may have been higher in December than in January/February. However, data on wind speed and atmospheric pressure, combined with direct field observations, do not support this as the storm in February was very strong and comparable to the heaviest storm in December. Second, the average condition or state of birds still breeding in January and February may be higher than the condition of birds in December. Indeed, it is likely that most of the birds in a poor condition/state abandoned their nest after the first storms, and that only birds in a good condition were present hereafter. Such birds may have been able to better resist the January and February snow storms, leading consequently to a lower impact of those storms. And third, snow storms may have indirectly affected nest survival through an effect of increased melt water, which could have killed some eggs and/or chicks by decreasing their temperature below a sustainable threshold (Varpe and Tveraa 2005). Nest sites that are filled with a layer of gravel are better protected from melt water than nests without gravel (Moreno et al. 1995, S.-H. Lorentsen unpubl. data). Thus, nests without gravel might have failed after the first snow storm and only the “best” nest sites (in the context of a snow storm) may have survived, leading to a higher nest survival during snow storm events in January/February.

At present, we cannot disentangle between these nonexclusive hypotheses, that is, higher sensitivity to snow storm when fasting vs. within-season selection toward “high-quality birds” (Wilson and Nussey 2010) or “high-quality nest sites”, and further studies are needed.

### Snow storm, oceanographic and individual conditions

The previous “within-season selection” hypothesis stresses out the importance of considering individual characteristics to better assess population responses to extreme weather events. Relatively few studies have investigated individual heterogeneity in their response to climatic fluctuations in general or weather extremes in particular (but see Coulson et al. 2001; Lewis et al. 2009 for examples). However, it is critically important to understand interplay between environmental factors and individual heterogeneity as a differential impact on individuals will affect population structure, and hence population dynamics and viability (Coulson et al. 2001; Benton et al. 2006). Such individual heterogeneity in face of extreme events is supported by our results indicating that chicks in a poor condition were more likely to die during a snow storm than chicks in a good condition. Such individual differences likely represent differences among parents in their ability to forage and feed their chick. State-dependent responses in parental care are indeed well known in long-lived seabirds, including the Antarctic petrel (Tveraa et al. 1997; Varpe et al. 2004).

On an interannual scale, a snow storm may have exacerbated effects if it occurs in a year of low food availability. Our results indicate that even without the storms, the breeding success in 2011/2012 would have been very low. It could be that snow storms in 2011/2012 had such a dramatic effect on nest survival because birds (adults or chicks) were, on average, in a poor condition. Unfortunately, no data were available to test this hypothesis. To understand whether snow storms and oceanographic conditions can have synergistic effects on breeding success needs further work and ideally longer data time series on body condition and breeding success, snow storm occurrence/severity, and availability of marine resources.

## Conclusion

Summer snow storms had a strong and significant effect on Antarctic petrel nest survival and are an important driver of annual breeding success. This emphasizes the importance of direct effects of extreme weather conditions on seabird vital rates. Across Antarctica, climatic conditions have shown significant changes over the last decades, and these changes have been more or less pronounced depending on the area considered (Turner et al. 2005). Even if warming is currently focused around the Antarctic Peninsula, climate models predict positive temperature trends all over Antarctica in the 21st century (Solomon et al. 2007). Moreover, most models predict a concurrent increase in precipitation (Tebaldi et al. 2006; Genthon et al. 2009) associated with a decrease in pressure (Turner et al. 2005) and an increase in wind speeds (Thompson and Solomon 2002; Turner et al. 2005). Such trends may lead to stronger and/or more frequent extreme weather events, including snow storms. We found no evidence that snow storm frequency increased in the past three decades in our study area, but additional data would be needed to draw conclusions about snow storm severity. In comparison, an increase in snow storm severity and frequency seems already ongoing along the nearby Antarctic Peninsula, at least in spring, with important ecological effects (McClintock et al. 2008).

Snow storms may have amplified effects in years of low food availability, which could be affected by future environmental changes. Antarctic petrels rely strongly on Antarctic krill, the dominant prey of their diet (Lorentsen et al. 1998). If Antarctic krill abundance was to decline in response to the warming and acidification of the southern ocean (Atkinson et al. 2004; Kawaguchi et al. 2013), snow storms occurring during the breeding season could threaten the long-term viability of some Antarctic seabirds populations even at their current frequency and severity level.

## Supporting Information

**Appendix S1** Frequency and duration of snow storm events in the past decades. **Figure S1. (**A**)** Time series from Troll and Neumayer weather stations for the summer 2011–2012 for Wind Speed (left *Y*-axis) measurements and Atmospheric Pressure (right *Y*-axis). (B) Frequency distribution atmospheric pressure measurements at Neumayer Station (40 m above sea level) from December to February each year, pooled over the period 1981–2013. **Table S1.** (A) Summary of the time series of weather data used in our analyses from three research stations. (B) Values of the cross-correlation coefficients calculated when comparing times series of weather data (wind speed and atmospheric pressure) at Troll vs. Neumayer stations. (C) Estimated number of storm days per breeding season, from 1981 to 2014 (estimated number of occurences in parantheses), corresponding to the data used in Fig. 3.
