# Supplementary material for: Demographic effects of extreme weather events: snow storms, breeding success, and population growth rate in a long-lived Antarctic seabird
Source: Ecol Evol. 2014 Dec 23;5(2):314–25. doi: 10.1002/ece3.1357 (PMC4314264; doi:10.1002/ece3.1357)
Supplement: Supplementary file 1 [file ece30005-0314-sd1.docx]

**Supplementary material**

**Appendix S1. Frequency and duration of snow storm events in the past decades**

*Step 1. Are weather conditions correlated at large geographical scale? Comparison between Neumayer and Troll weather stations.*

There is no long term weather monitoring at Svarthamaren. In order to estimate the frequency of snow storm events during the past decades, we had to examine long term weather datasets from some of the closest stations in Dronning Maud Land. We assumed that strong weather events such as snow storms and blizzards usually occur at spatial scales of hundreds of kilometers, such that weather conditions at a given location can be determined by examining the weather conditions from another neighboring location.

In order to assess the validity of this assumption, we used meteorogical data from Troll (72°00’S, 2°32′E; 1270 m a.s.l. and *ca*. 90 km away from Svarthamaren) and Neumayer (70°40’S, 8°16’W; *ca*. 40 m a.s.l. and 500 km away from Svarthamaren) research stations (Table S1-a). Available overlapping weather data from Neumayer cover the period from 1982 to 2013, and were obtained for from *www.pangaea.de* (König-Langlo & Loose, 2007). Data from Troll were obtained from the Norwegian Polar Institute, and covered the period 2008-2013. We used wind speed (m.s^-1^) at 10m above ground and atmospheric pressure (hPa) as indicators of stormy weather conditions during the austral summer (December to February), and averaged values per day in order to obtain the same temporal resolution for both time series. Values for atmospheric pressure at Troll were corrected in order to take altitude difference into account (y’=y+140hPa).

Then we tested for temporal correlation between the weather observations at Svarthamaren /Troll vs. Neumayer. Using the function *acf* from package *stats* in R.2.14.2 (R Development Core Team, 2010), we calculated cross-correlations between time series of daily average wind speed of both stations for the period of overlapping monitoring (i.e. 2008 to 2013). We tested for time lags of up to 6 days, in order to confirm that the weather observations at Neumayer could reliably depict the weather pattern experienced at Troll/Svarthammaren during the same period. All cross-correlation coefficients were highest for a lag of 0 day and ranged from 0.50 to 0.70 for wind speed and from 0.81 to 0.87 for atmospheric pressure (Table S1-b).

Weather conditions at Troll and Neumayer generally fluctuated similarly over the period covered, thereby confirming that conditions at Neumayer could realistically be used as proxies to describe weather conditions at Troll and our study site.

*Step 2. Can pressure and wind speed data be used to detect storm events at our study site?*

We visually compared the time series available from Troll and Neumayer for seasons 2011-2012 and 2012-2013, during which we had a detailed record of the occurrence of severe storms at Svarthammaren from early December to late February (Fig. S1-a).

It has been observed at Neumayer station that snow starts to drift at wind speeds of 6-12 m.s^1^ depending on surface conditions (König-Langlo & Loose, 2007). Thus, we used 12 m.s^-1^ as a conservative threshold for daily average wind speed above which we could assume that snow would drift heavily and potentially accumulate on nests. For atmospheric pressure, we used the 5% percentile from the frequency distribution of all summer measurements from all years (975 hPa; Fig. S1-b) as a threshold value.

All four snow storm events observed *in situ* at Svarthammaren in 2011-2012 were associated with peaks in wind speed > 12 m.s^-1^, combined with lows in atmospheric pressure < 975 hPa, that lasted for at least 24 h (Fig. S1-a). Therefore, we considered that wind speed and pressure could be used as indicators of the occurrence of snow storms.

*Step 3. Reconstructing the snow storm history over the last three decades.*

In a last step, we used these pressure and wind speed threshold values to determine the likely occurrence and duration of severe storm events in the past. Accordingly, we automatically assigned an episode of “severe storm” to any given period that matched the above-described pattern, i.e. when both wind speed and atmospheric pressure values were respectively above and below these thresholds. Because very temporary (i.e., 24 h or less) lows in atmospheric pressure data were very common in the time series, and would have led to a likely high number of false positives, we decided to consider only periods during which atmospheric pressure was under the threshold value for at least two consecutive days. This allows us to reconstruct the occurrences and duration of snow storm events during the Antarctic petrel breeding season at Svarthamaren for the past 32 years (Table S1-c). Even if the number of storms might have been higher, we do not expect our potential underestimation to be biased but rather consistent throughout the years, and it should thus not influence analyses focused on inter-annual differences in storm occurrences.


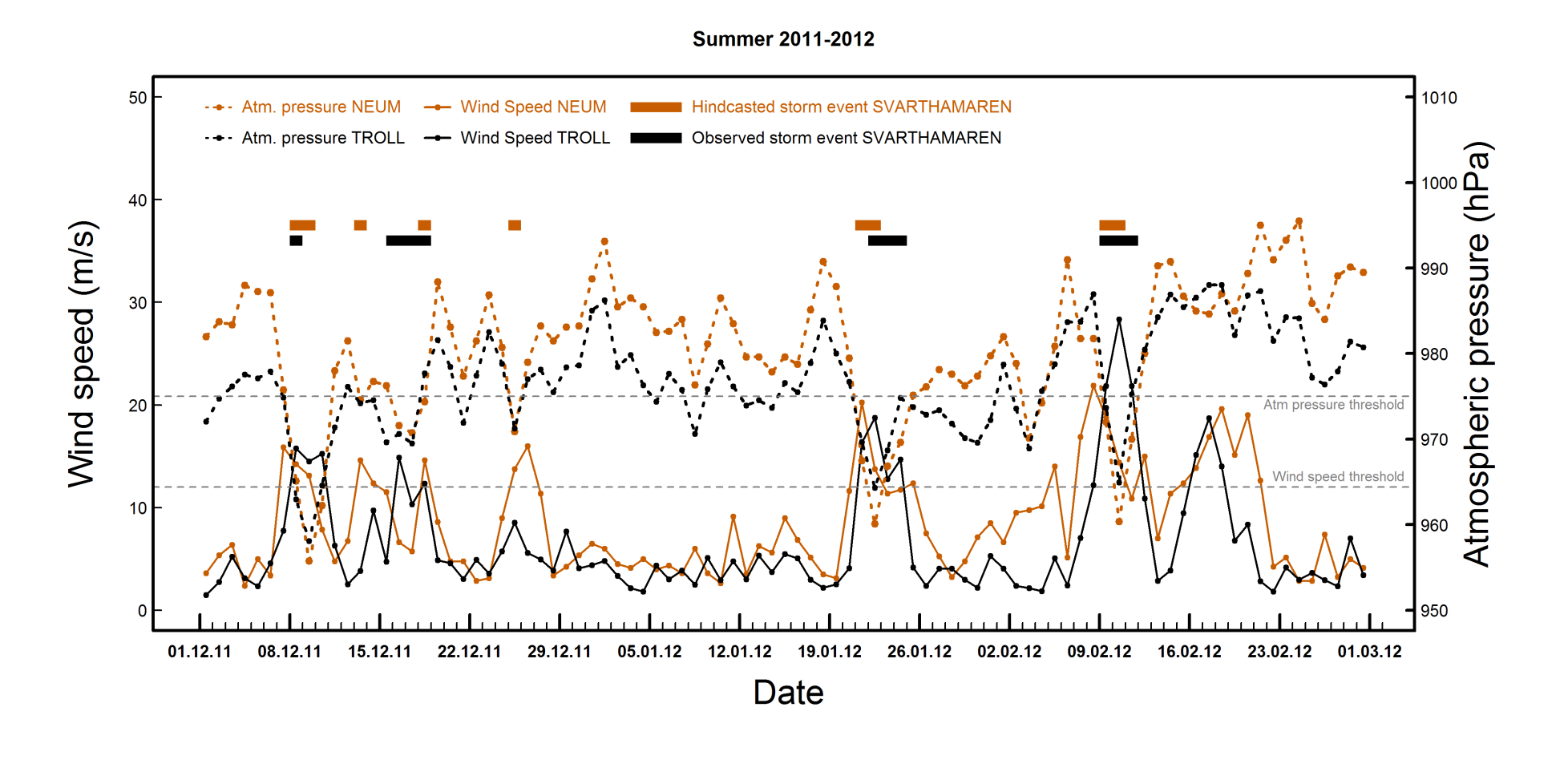


**Figure S1-a.** Time series from Troll and Neumayer weather stations for the summer 2011-2012 for Wind Speed (left Y-axis) measurements and Atmospheric Pressure (right Y-axis). Atmospheric pressure values from Troll were adjusted to compensate for altitude difference between the two stations (y’=y+140hPa). Episodes of known storm events at Svarthammaren (blue rectangles) and estimated storm events based on weather data from Neumayer (see Appendix S1 for details; orange rectangles), are also indicated. Dashed horizontal lines show the upper wind speed and lower atmospheric pressure thresholds used for determining periods of stormy conditions.


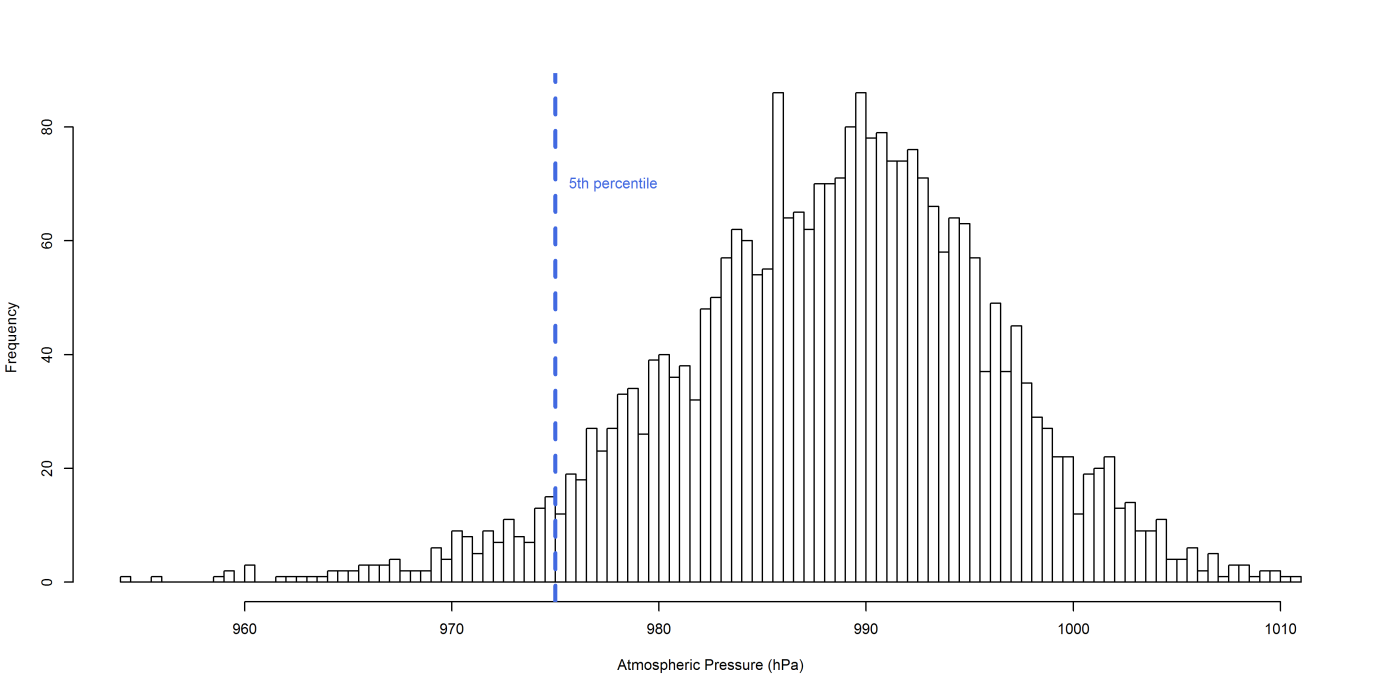


**Figure S1-b.** Frequency distribution atmospheric pressure measurements at Neumayer Station (40 m above sea level) from December to February each year, pooled over the period 1981-2013. The lower 5% percentile (blue dashed line) was used as a threshold to detect unusually low pressure measurements in the time series (see Appendix S1).

**Table S1-a.** Summary of the time series of weather data used in our analyses from three research stations.

| **Station** | **Period**  **covered** | **Variables**  **used** | **Measurement**  **frequency** |
| --- | --- | --- | --- |
| Neumayer | Dec 1982 to Mar 2013 | Wind, Atmospheric pressure | Every 6h |
| Troll | Dec 2008 to Mar 2013 | Wind, Atmospheric pressure | Every 3h |
| Tor | Dec 2011 to Feb 2013 | Field observations of storm events | Daily |

**Table S1-b.** Values of the cross-correlation coefficients calculated when comparing times series of weather data (wind speed and atmospheric pressure) at Troll vs. Neumayer stations. Asterisks (*) indicate that the correlation coefficient was significantly different from 0 (p < 0.001, N = 10000 permutations). The highest correlation coefficient value for each season/variable is in bold.

|  | **Lag** | **2008-2009** | **2009-2010** | **2010-2011** | **2011-2012** | **2012-2013** |
| --- | --- | --- | --- | --- | --- | --- |
| **Atmospheric Pressure** | -6 day | 0.37* | 0.29 | -0.05 | 0.08 | 0.31 |
|  | -5 day | 0.32* | 0.24 | 0 | 0.02 | 0.32 |
|  | -4 day | 0.37* | 0.31 | 0.27 | -0.01 | 0.33 |
|  | -3 day | 0.48* | 0.31 | 0.46* | 0.08 | 0.4 |
|  | -2 day | 0.51* | 0.31 | 0.46* | 0.28 | 0.47* |
|  | -1 day | 0.61* | 0.54* | 0.58* | 0.59* | 0.63* |
|  | **0 day** | **0.85*** | **0.87*** | **0.84*** | **0.86*** | **0.81*** |
|  | 1 day | 0.66* | 0.63* | 0.61* | 0.66* | 0.66* |
|  | 2 day | 0.46* | 0.33 | 0.37* | 0.3 | 0.44* |
|  | 3 day | 0.44* | 0.27 | 0.38* | 0.16 | 0.34 |
|  | 4 day | 0.36* | 0.28 | 0.26 | 0.18 | 0.24 |
|  | 5 day | 0.26 | 0.19 | 0.05 | 0.25 | 0.25 |
|  | 6 day | 0.29 | 0.13 | 0 | 0.32 | 0.3 |
| **Wind Speed** | -6 day | 0.05 | 0.08 | -0.11 | 0 | -0.05 |
|  | -5 day | 0.26 | 0.09 | -0.04 | -0.03 | -0.02 |
|  | -4 day | 0.26 | 0.17 | 0.12 | 0.02 | 0.19 |
|  | -3 day | 0.38 | 0.24 | 0.18 | 0.27 | 0.32 |
|  | -2 day | 0.45* | 0.2 | 0.22 | 0.46* | 0.35 |
|  | -1 day | 0.42* | 0.24 | 0.46* | 0.6* | 0.49* |
|  | **0 day** | **0.54*** | **0.5*** | **0.7*** | **0.62*** | **0.63*** |
|  | 1 day | 0.37 | 0.43* | 0.52* | 0.43* | 0.54* |
|  | 2 day | 0.29 | 0.24 | 0.23 | 0.3 | 0.4 |
|  | 3 day | 0.18 | 0.18 | 0.07 | 0.16 | 0.14 |
|  | 4 day | 0.08 | 0.12 | -0.05 | 0.07 | -0.13 |
|  | 5 day | 0 | 0.06 | -0.16 | 0 | -0.26 |
|  | 6 day | -0.19 | 0.03 | -0.25 | -0.03 | -0.23 |

**Table S1-c.** Estimated number of storm days per breeding season, from 1981 to 2014 (estimated number of occurences in parantheses), corresponding to the data used in Fig. 3. Only storms that occurred before the annual colony monitoring (last half of January) were taken into account. Storm occurrence/duration was inferred from weather data (wind speed and atmospheric pressure) available from Neumayer research station (see Appendix S1 for details), or from direct field observations. When available, nest count data from *in situ* monitoring were indicated.

| **Summer season** | **Cumulated nb of storm days (occurrences)** | **Estimated**  **nest count ^a^** |
| --- | --- | --- |
| 1981-1982 | 8 (2) | n/a |
| 1982-1983 | 0 | n/a |
| 1983-1984 | 0 | n/a |
| **1984-1985** | 0 | **207480 ^c^** |
| 1985-1986 | 3 (2) | n/a |
| 1986-1987 | 0 | n/a |
| 1987-1988 | 0 | n/a |
| 1988-1989 | 0 | n/a |
| 1989-1990 | 0 | **171990 ^c^** |
| 1990-1991 | 0 | n/a |
| **1991-1992 ^b^** | 2 (2) | **115855** |
| **1992-1993 ^b^** | 3 (1) | **80139** |
| **1993-1994** | 0 | **163506** |
| **1994-1995** | 3 (1) | **47016** |
| 1995-1996 | 2 (1) | n/a |
| **1996-1997** | 0 | **111111** |
| **1997-1998** | 0 | **68395** |
| 1998-1999 | 5 (3) | n/a |
| 1999-2000 | 3 (2) | n/a |
| **2000-2001** | 0 | **118800** |
| 2001-2002 | 2 (2) | n/a |
| 2002-2003 | 0 | n/a |
| 2003-2004 | 1 (1) | n/a |
| 2004-2005 | 0 | n/a |
| 2005-2006 | 0 | n/a |
| 2006-2007 | 0 | n/a |
| 2007-2008 | 0 | n/a |
| 2008-2009 | 0 | n/a |
| 2009-2010 | 0 | n/a |
| 2010-2011 | 2 (1) | n/a |
| **2011-2012 ^b^** | 8 (3) | **27233**^d^ |
| **2012-2013 ^b^** | 0 | **72800** |
| **2013-2014 ^b^** | 0 | **47986** |

^a^ Total number of nests estimated for the whole colony through systematic monitoring of plots (Lorentsen et al., 1993)

^b^ Occurrence and duration of storm events for those years are based on detailed field records *in situ* at Svarthamaren.

^c^  Values from Mehlum et al. (1985) and Røv (1991)

^d^ Value corrected in order to compensate for the fact that monitoring took place later than usual on that year (i.e., on 5 February instead of 25 January). We therefore estimated the likely number of active nests on 25 January using an average daily nest survival value of 0.97 (based on estimates from Model 1, Table 1).
